# Poplar Rows in Temperate Agroforestry Croplands Promote Bacteria, Fungi, and Denitrification Genes in Soils

**DOI:** 10.3389/fmicb.2019.03108

**Published:** 2020-01-22

**Authors:** Lukas Beule, Ena Lehtsaar, Marife D. Corre, Marcus Schmidt, Edzo Veldkamp, Petr Karlovsky

**Affiliations:** ^1^Molecular Phytopathology and Mycotoxin Research, Faculty of Agricultural Sciences, University of Goettingen, Göttingen, Germany; ^2^Soil Science of Tropical and Subtropical Ecosystems, Faculty of Forest Sciences and Forest Ecology, University of Goettingen, Göttingen, Germany

**Keywords:** temperate agroforestry cropland, alley-cropping, soil bacteria, soil fungi, N-cycling genes

## Abstract

Agroforestry, which is the integration of trees into monoculture cropland, can alter soil properties and nutrient cycling. Temperate agroforestry practices have been shown to affect soil microbial communities as indicated by changes in enzyme activities, substrate-induced respiration, and microbial biomass. Research exploring soil microbial communities in temperate agroforestry with the help of molecular tools which allow for the quantification of microbial taxa and selected genes is scarce. Here, we quantified 13 taxonomic groups of microorganisms and nine genes involved in N cycling (N_2_ fixation, nitrification, and denitrification) in soils of three paired temperate agroforestry and conventional monoculture croplands using real-time PCR. The agroforestry croplands were poplar-based alley-cropping systems in which samples were collected in the tree rows as well as within the crop rows at three distances from the tree rows. The abundance of Acidobacteria, Actinobacteria, Alpha- and Gammaproteobacteria, Firmicutes, and Verrucomicrobia increased in the vicinity of poplar trees, which may be accounted for by the presence of persistent poplar roots as well as by the input of tree litter. The strongest population increase was observed for Basidiomycota, which was likely related to high soil moisture, the accumulation of tree litter, and the absence of tillage in the tree rows. Soil microorganisms carrying denitrification genes were more abundant in the tree rows than in the crop rows and monoculture systems, suggesting a greater potential for nitrate removal through denitrification, which may reduce nitrate leaching. Since microbial communities are involved in critical soil processes, we expect that the combination of real-time PCR with soil process measurements will greatly enhance insights into the microbial control of important soil functions in agroforestry systems.

## Introduction

Modern agroforestry systems (e.g., alley-cropping of crops and short-rotation trees) have been recognized as multifunctional systems that can reduce nitrate leaching, increase carbon sequestration, and increase pollination services (Kay et al., 2018). Likewise, the practice of agroforestry in Europe can enhance biodiversity and soil fertility relative to monoculture agriculture (Torralba et al., 2016), while also maintaining agricultural productivity (Pardon et al., 2018; Swieter et al., 2018) and food safety of small-grain cereals such as wheat (*Triticum aestivum*) and barley (*Hordeum vulgare*) (Beule et al., 2019b). In such systems, ecological interactions between crops and trees can yield greater overall resource-use efficiency if the positive interactions outweigh competitive effects (Cannell et al., 1996; van Noordwijk et al., 2015). For example, deep-rooting trees are able to take up leached nutrients from soil layers that are not accessible to crops (Allen et al., 2004; Wang et al., 2011). Depending on the age of the tree component, temperate agroforestry has shown to increase soil organic carbon (SOC) stocks and soil nutrient availability, especially close to the trees (Cardinael et al., 2015, 2019; Pardon et al., 2017). In order to account for the spatial heterogeneity within agroforestry systems, several studies applied transectal sampling strategies such as sampling in the crop rows at different distances from the tree rows (Cardinael et al., 2015; Pardon et al., 2017; Swieter et al., 2018).

Over the past 20 years, a number of studies investigated soil microorganisms in temperate agroforestry systems by using enzyme assays, substrate-induced respiration or microbial biomass determination (e.g., Seiter et al., 1999; Lee and Jose, 2003; Mungai et al., 2005; Udawatta et al., 2008, 2009; Rivest et al., 2013; Weerasekara et al., 2016; Sun et al., 2018; Beuschel et al., 2019). Agroforestry has been shown to increase functional diversity of enzyme activities (Seiter et al., 1999; Mungai et al., 2005; Udawatta et al., 2008, 2009; Unger et al., 2013; Weerasekara et al., 2016). In an alder (*Alnus rubra*)-sweet corn (*Zea mays*) alley-cropping system, active bacterial and fungal biomass in the corn row declined with increasing distance from the tree row (Seiter et al., 1999). In contrast to these results, Saggar et al. (2001) reported a strong suppression of soil microbial biomass by pine (*Pinus radiata*) trees planted in grassland. Increased fungi-to-bacteria ratios were reported in the tree row compared to the crop row of agroforestry systems (Beuschel et al., 2019) and the analysis of phospholipid fatty acids showed increased abundance of gram-positive, gram-negative and anaerobic soil bacteria in agroforestry as compared to cropland soil (Unger et al., 2013). Additionally, the integration of trees into agricultural fields decreased the metabolic quotient indicating a greater substrate-use efficiency of soil microorganisms (Rivest et al., 2013; Beuschel et al., 2019).

Molecular studies investigating microbial communities or functional genes in soils of temperate agroforestry systems are scarce. In an initial study, Udawatta et al. (2008) found that total soil-extractable DNA, used as a proxy for soil microbial biomass, was higher in agroforestry than in cropland and grassland but recommended the use of taxon-specific PCR assays to assess differences in soil microbial communities between the tree and crop rows. Their suggestion has only recently been implemented in a study of temperate agroforestry cropland and grassland which showed increased fungi-to-bacteria ratio under trees, and alterations of ammonium-oxidizing populations (Beule et al., 2019a). The investigation of genes involved in soil-N cycling in agricultural systems is important as these genes reveal the genetic potential to control N fluxes such as nitrous oxide (N_2_O) emissions. In a recent large-scale study, Banerjee et al. (2016) investigated soil bacterial communities in Canadian agroforestry systems using amplicon sequencing and concluded that agroforestry affects the abundance of certain bacterial taxa and supported bacterial growth, in general, but did not promote bacterial diversity (Banerjee et al., 2016).

The aim of this study was to investigate spatial variation in the abundances of major groups of soil bacteria and fungi and soil-N-cycling genes (N_2_ fixation, nitrification, and denitrification) in temperate agroforestry as compared to conventional monoculture cropland. We accounted for the predicted spatial heterogeneity within agroforestry systems and sampled along transects spanning from the center of the tree row to the center of the crop row. We hypothesized that the trees in the agroforestry systems will promote the abundance of soil bacteria, fungi, and N-cycling genes due to improved soil properties from tree litter inputs and absence of tillage in the tree rows.

## Materials and Methods

### Study Sites and Experimental Design

**FIGURE 1:**
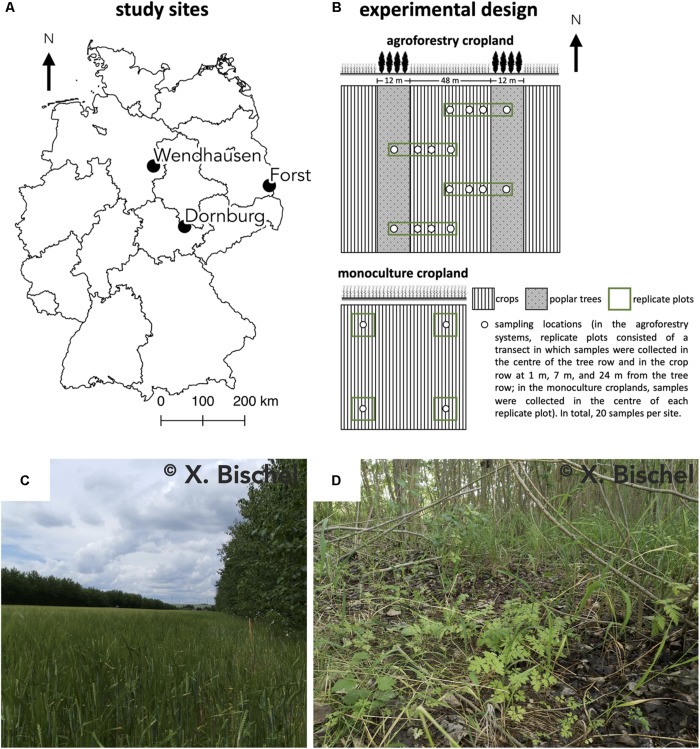
**(A)** Locations of the three study sites in Germany, **(B)** the experimental design at each site, **(C)** the alley-cropping agroforestry system, and **(D)** the herbaceous layer under the trees of the agroforestry system at the Dornburg site. Calcaric Phaeozem soil in Dornburg, Gleyic Cambisol soil in Forst, and Vertic Cambisol soil in Wendhausen.

Our study was conducted at three sites where conventional monoculture cropland has been converted into an alley-cropping agroforestry system (Figure 1). Each of these agroforestry cropland systems was compared to an adjacent monoculture cropland system representative of the management prior to agroforestry conversion. The three study sites in Germany were near Dornburg, Thuringia with a Calcaric Phaeozem soil, near Forst, Brandenburg with a Gleyic Cambisol soil, and near Wendhausen, Lower Saxony with a Vertic Cambisol soil (Figure 1A and Table 1). Hereafter, we refer to these study sites by their soil types. The alley-cropping agroforestry systems were established between 2007 and 2010 (Table 1): 12-m wide poplar rows planted with poplar cuttings (clone Max1; *Populus nigra* × *P. maximowiczii*) were interspersed with 48-m wide crop rows in a North-South orientation (Figures 1B,C). The poplar cuttings were planted by hand using a dibble bar. The first harvest of the aboveground biomass of the trees was conducted in January 2015 at the Calcaric Phaeozem site, in February 2014 at the Gleyic Cambisol site, and in January 2014 at the Vertic Cambisol site (Table 1). The crop rows of the agroforestry systems were managed in the same way as their corresponding adjacent monoculture cropland systems (identical crops, pesticide and herbicide applications, harvesting methods, and fertilization period and rates). The crop rotations at the three study sites included maize (*Zea mays*), summer and winter barley (*H. vulgare*), winter oilseed rape (*Brassica napus*), and winter wheat (*T. aestivum*) (Table 1). Fertilization rates were typical of the farmers’ practice at these sites (Table 1), and fertilization is commonly carried out in spring to monoculture croplands and agroforestry crop rows. The agroforestry tree rows were not fertilized as customary in temperate agroforestry practices (Tsonkova et al., 2012).

**TABLE 1:** Site characteristics and management at the three study sites of paired temperate agroforestry and monoculture cropland.

**Study site**	**Dornburg**	**Forst**	**Wendhausen**
Location	51°00′40′′N, 11°38′46′′E	51°47′11′′N, 14°38′05′′E	52°20′00′′N, 10°37′55′′E
Soil type	Calcaric Phaeozem	Gleyic Cambisol	Vertic Cambisol
Mean annual air temperature (1981–2010)	9.9 ± 0.1°C^a^	9.6 ± 0.2°C^b^	9.6 ± 0.2°C^c^
Mean annual precipitation (1981–2010)	608 ± 21 mm^a^	568 ± 21 mm^b^	637 ± 23 mm^c^
Meters above sea level	289 m	67 m	82 m
Year of agroforestry system establishment	2007	2010	2008
Harvest(s) of the aboveground tree biomass of the agroforestry system	January 2015	February 2015, March 2018	January 2014
Crop rotation (2016 – 2017 – 2018 – 2019)	Summer barley– winter oilseed rape – winter wheat – summer barley	Winter wheat – winter barley – maize – summer barley	Winter oilseed rape – winter wheat – winter wheat – maize
Fertilization rates in 2018 (kg N – P – K ha^–1^ yr^–1^)	213.0 – 0.0 – 0.0	64.0 – 19.8 – 47.1	166.0 – 0.0 – 116.2

*Mean ± standard error during 1981–2010; ^*a*^climate station at Jena (station ID: 2444), ^*b*^Cottbus (station ID: 880), and ^*c*^Braunschweig (station ID: 662) of the German Meteorological Service.*

At every site, four replicate plots were established in both the agroforestry and monoculture cropland system (Figure 1B). In the agroforestry systems, replicate plots consisted of a transect spanning from the center of the 12-m wide tree row to the center of the 48-m wide crop row (orthogonal to the North-South orientation of the tree rows). Within each of these transects, soil samples were collected in the center of the tree row (between the poplar trees at approximately at 1 m distance from the trunks) as well as at in the crop row at 1, 7, and 24 m distance from the tree row. Altogether, resulting in four samples per replicate plot of the agroforestry systems (Figure 1B). In the corresponding monoculture croplands, samples were collected in the center of each replicate plot (Figure 1B), resulting in four samples for the monoculture system per site. In total, 20 samples per site. Soil samples were collected in both management systems at each site in spring 2019, prior to fertilization in that year.

### Soil Sampling and DNA Extraction

Soil sampling was conducted on 4 March 2019 in the Calcaric Phaeozem (291 days post fertilization), 1 April 2019 in the Gleyic Cambisol (363 days post fertilization), and 26 March 2019 in the Vertic Cambisol (316 days post fertilization). At each sampling location in each replicate plot, four 250 cm^3^ soil samples of the top 5-cm depth were collected. One of these samples was used to determine the water-filled pore space (WFPS), the other three were pooled and thoroughly homogenized immediately after sampling to obtain one composite soil sample per plot for DNA extraction. While still in the field an aliquot of approximately 20 g soil of each composite sample was transferred to a sterile 15-mL polypropylene Falcon tube and frozen at −20°C. Upon arrival at the laboratory, the samples were stored at −20°C. For DNA extraction, soil samples were freeze-dried for 72 h and homogenized using a swing mill (MM400, Retsch, Haan, Germany) for one minute at 25 Hz. DNA from 50 mg of finely ground soil was extracted as described in Beule et al. (2019b). Quantity and quality of the extracted DNA were assessed on 1.7% (w/v) agarose gels stained with ethidium bromide. DNA extracts were stored at −20°C until analysis.

### Real-Time PCR Assays

We quantified 13 taxonomic groups of soil microorganisms: total bacteria, Acidobacteria, Actinobacteria, Alphaproteobacteria, Bacteriodetes, Betaproteobacteria, Firmicutes, Gammapro- teobacteria, Gemmatimonadetes, Verrucomicrobia, total fungi, Ascomycota, and Basidiomycota as well as nine genes involved in N cycling: *nifH* for N_2_ fixation, ammonium-oxidizing archaea (AOA) *amoA* and ammonium-oxidizing bacteria (AOB) *amoA* for the oxidization of ammonia, *napA* and *narG* for the reduction of nitrate, *nirK* and *nirS* for the reduction of nitrite, and *nosZ* clade I and II the reduction of N_2_O. The primers used are listed in Supplementary Table 1.

Standard curves for real-time PCR (qPCR) assays were generated in two replicates using 1:3 or 1:10 serial dilutions of quantified PCR products dissolved in 0.5X TE buffer (5 mM Tris/HCl, 0.5 mM EDTA, pH 8.0). DNA from organisms carrying the target genes (Supplementary Table 1) was extracted using a CTAB protocol (Brandfass and Karlovsky, 2008) and used as a template for PCR generating standards. All qPCRs were carried out in a CFX384 Thermocycler (Bio-Rad, Rüdigheim, Germany) in 384-well microplates. Amplifications of microbial groups and N_2_ fixation gene *nifH* were performed with 1:50 dilutions of the DNA extracts in 4 μl reaction volume containing 3 μl mastermix [double-distilled H_2_O, reaction buffer (Supplementary Table 2); 1.5, 2.0 or 2.5 mM MgCl_2_ (Supplementary Table 2); 100 μM of each deoxyribonucleoside triphosphate (Bioline, Luckenwalde, Germany); 0.3, 0.5 or 0.75 μM of each primer (Supplementary Table 2); 1 mg/ml bovine serum albumin; 0.03 u DNA Polymerase (Supplementary Table 2)] and 1 μl template DNA solution or double-distilled H_2_O for negative controls. The thermocycling conditions of all quantified microbial groups are reported in Supplementary Table 2. Genes involved in nitrification and denitrification were amplified according to Beule et al. (2019b). Melting curves were obtained by heating the samples to 95°C for 60 s and cooling to 55°C for 60 s followed by a temperature increase from 55°C to 95°C by 0.5°C per step with continuous fluorescence measurement.

### Determination of Soil Properties

Soil samples for the determination of soil properties were collected in autumn 2016. For the determination of pH, SOC, total N, texture, exchangeable bases, and effective cation exchange capacity (ECEC), soil samples were air-dried and sieved to <2 mm. Soil pH was determined at a soil-to-distilled water ratio of 1:4 (w/v). SOC [after acid-fumigation (Harris et al., 2001)] and total N were determined using a CN analyzer (Elementar Vario EL, Elementar Analysis Systems GmbH, Hanau, Germany). ECEC, including exchangeable bases, was analyzed by an inductively coupled plasma-atomic emission spectrometer (ICP-OES, iCAP 6300 Duo View ICP Spectrometer, Thermo Fischer Scientific GmbH, Dreieich, Germany), after percolation of the soil with unbuffered 1 M NH_4_Cl solution. Soil texture was determined by applying the pipette method after removing organic matter, iron oxide, and carbonates (Kroetsch and Wang, 2008). WFPS was determined from oven-drying of fresh soil (collected on the same day that samples for DNA extraction were collected) for 24 h at 105°C using a particle density of 2.65 g cm^–3^ as well as the bulk density measured on site via the core method (Blake and Hartge, 1986).

### Statistical Analysis

Each parameter was tested for normality of distribution (Shapiro–Wilk’s test) and homogeneity of variances (Levene’s test). Differences in soil properties among the sampling locations within the agroforestry cropland (the tree row, 1, 7, and 24 m from the tree row within the crop row) and the monoculture cropland within each soil type were tested using one-way analysis of variance (ANOVA) with Tukey’s honestly significant difference (HSD) test or Kruskal–Wallis test with multiple comparison extension. Differences in log_10_-transformed gene abundances of both microbial groups and N-cycling genes among the sampling locations within the agroforestry cropland and the monoculture cropland within each soil type were determined using one-way ANOVA with Tukey’s HSD test. Relationships among the abundances of microbial groups, N-cycling genes and soil properties were analyzed across all replicate plots of agroforestry and monoculture croplands in all three soil types using Spearman’s rank correlation test. Statistical significance was considered at *p* < 0.05. All statistical analyses were performed in R version 3.4.3.

## Results

### Soil Bacterial and Fungal Populations

**FIGURE 2:**
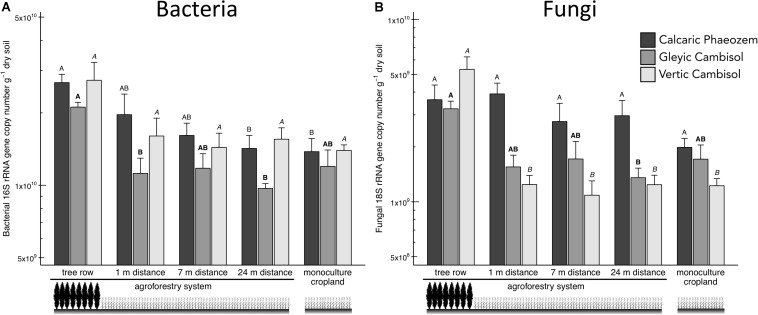
**(A)** Bacterial 16S rRNA and **(B)** fungal 18S rRNA gene abundance in soils of three paired temperate agroforestry and monoculture cropland systems. Means (bars are standard errors; *n* = 4) with different uppercase letters of the same font indicate significant differences among the sampling locations (the tree row, 1, 7, and 24 m within the crop row of the agroforestry and the monoculture croplands) within one soil type (one-way ANOVA with Tukey’s HSD test of log_10_-transformed data at *p* < 0.05).

Total soil bacteria in the Calcaric Phaeozem and total soil fungi in the Gleyic Cambisol were more abundant in the tree row than in the middle of the crop row (24 m distance from the tree row) of the agroforestry and the monoculture cropland (*p* ≤ 0.039) (Figures 2A,B). In the Vertic Cambisol, total soil fungi were greater in the tree row than in the crop row and the monoculture cropland (*p* < 0.001) (Figure 2A). The fungi-to-bacteria ratio was not affected by the practice of agroforestry (Supplementary Figure 1).

**FIGURE 3:**
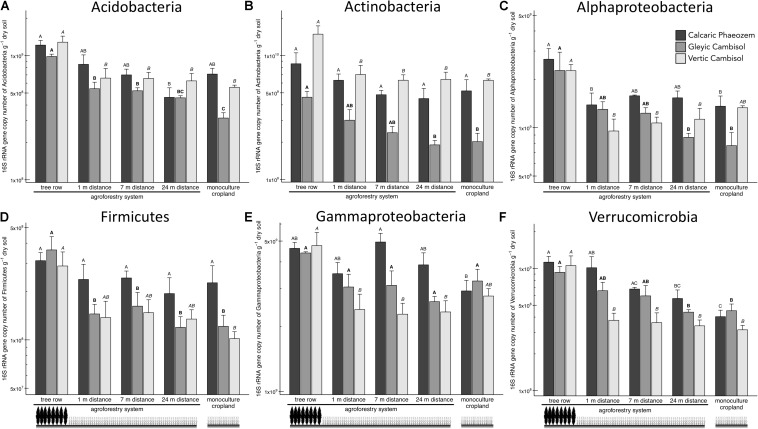
16S rRNA gene abundance of six bacterial groups [**(A)** Acidobacteria, **(B)** Actinobacteria, **(C)** Alphaproteobacteria, **(D)** Firmicutes, **(E)** Gammaproteobacteria, and **(F)** Verrucomicrobia] in soils of three paired temperate agroforestry and monoculture cropland systems. Means (bars are standard errors; *n* = 4) with different uppercase letters of the same font indicate significant differences among the sampling locations (the tree row, 1, 7, and 24 m within the crop row of the agroforestry and the monoculture croplands) within one soil type (one-way ANOVA with Tukey’s HSD test of log_10_-transformed data at *p* < 0.05).

Acidobacteria, Actinobacteria, Alpha- and Gamma proteobacteria, Firmicutes, and Verrucomicrobia showed a general pattern of greater abundance in the tree rows than the crop rows and/or the monoculture croplands (Figure 3). For example, in the Vertic Cambisol, Acidobacteria and Actinobacteria abundances were 2.0 to 2.4 times greater in the tree row than in the crop row and the monoculture cropland (*p* ≤ 0.035) (Figures 3A,B). Similarly, gene copies of Actinobacteria and Alphaproteobacteria in the Gleyic Cambisol were 2.3 to 2.9 times greater in the tree row than in middle of the crop row and the monoculture croplands (*p* ≤ 0.023) (Figures 3B,C). The strongest effects were observed for Verrucomicrobia: in all three soil types, Verrucomicrobia were more abundant in the tree row of the agroforestry croplands than in the monoculture croplands (*p* ≤ 0.020) (Figure 3F). The establishment of agroforestry did not alter the abundances of Bacteriodetes, Betaproteobacteria, and Gemmatimonadetes (Supplementary Figure 2).

**FIGURE 4:**
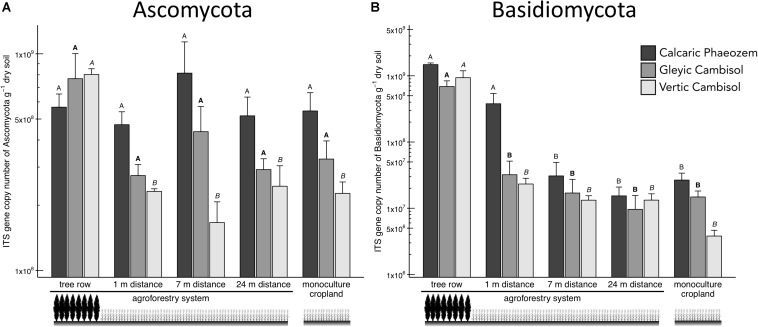
Internal transcribed spacer (ITS) gene abundance of fungal groups [**(A)** Ascomycota and **(B)** Basidiomycota] in soils of three paired temperate agroforestry and monoculture cropland systems. Means (bars are standard errors; *n* = 4) with different uppercase letters of the same font indicate statistically significant differences among the sampling locations (the tree row, 1, 7, and 24 m within the crop row of the agroforestry and the monoculture croplands) within one soil type (one-way ANOVA with Tukey’s HSD test of log_10_-transformed data at *p* < 0.05).

Two major soil fungal groups, Ascomycota and Basidiomycota, were also promoted in the tree rows of the agroforestry croplands (Figure 4). In particular, Ascomycota in the Vertic Cambisol and Basidiomycota in both Cambisol soils showed greater abundance in the tree row than in the crop row of the agroforestry and monoculture croplands (*p* ≤ 0.003) (Figures 4A,B). Likewise, Basidiomycota in the Calcaric Phaeozem were 12 to 96 times more abundant in the tree row and at 1-m crop row than at 7-m and 24-m crop row and the monoculture cropland (*p* ≤ 0.001) (Figure 4B).

### Genes Involved in Soil-N Cycling

**FIGURE 5:**
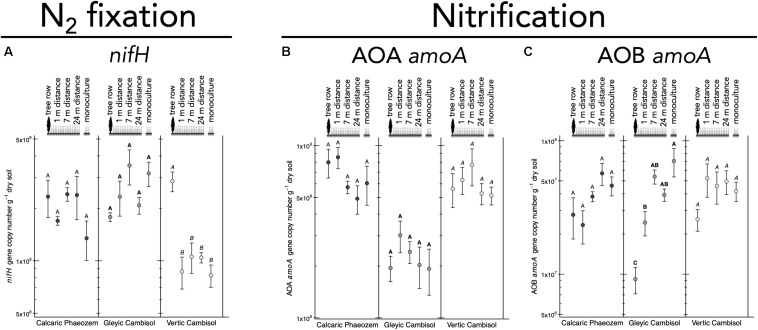
Gene abundance of **(A)** *nifH*, **(B)** AOA *amoA*, and **(C)** AOB *amoA* in soil of three paired temperate agroforestry and monoculture cropland systems. Means (bars are standard errors; *n* = 4) with different uppercase letters indicate statistically significant differences among the sampling locations (the tree row, 1, 7, and 24 m within the crop row of the agroforestry and the monoculture croplands) within one soil type (one-way ANOVA with Tukey’s HSD test of log_10_-transformed data at *p* < 0.05). AOA = ammonia-oxidizing archaea, AOB = ammonia-oxidizing bacteria.

**FIGURE 6:**
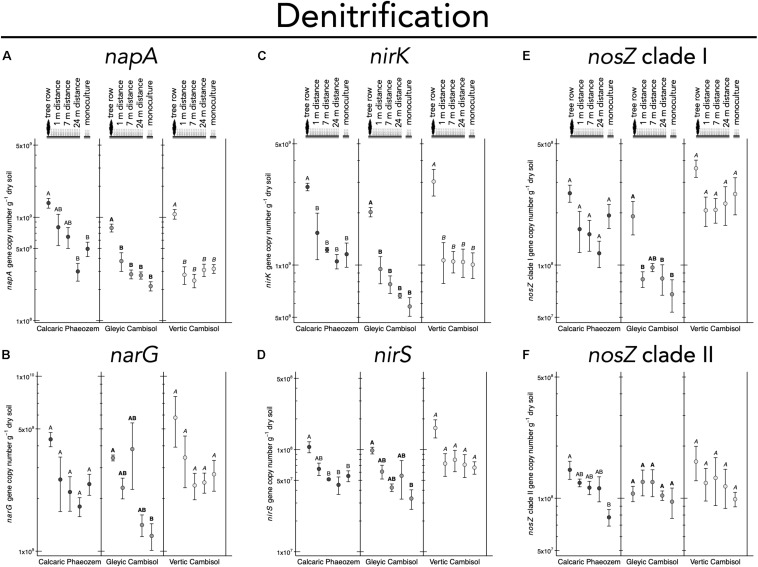
Gene abundance of **(A)** *napA*, **(B)** *narG*, **(C)** *nirK*, **(D)** *nirS*, **(E)** *nosZ* clade I, and **(F)** *nosZ* clade II in soil of three paired temperate agroforestry and monoculture cropland systems. Means (bars are standard errors; *n* = 4) with different uppercase letters indicate statistically significant differences among the sampling locations (the tree row, 1, 7, and 24 m within the crop row of the agroforestry and the monoculture croplands) within one soil type (one-way ANOVA with Tukey’s HSD test of log_10_-transformed data at *p* < 0.05).

N_2_ fixation gene *nifH* was 2.7 to 3.5 times more abundant in the tree row than in the crop row of the agroforestry and monoculture croplands in the Vertic Cambisol (*p* ≤ 0.019) (Figure 5A). In contrast, in the Gleyic Cambisol, AOB *amoA* gene abundance was lower in the tree row than in the crop row of the agroforestry and the monoculture croplands (*p* ≤ 0.020) (Figure 5C). Microorganisms harboring genes involved in denitrification were generally more abundant in the tree rows than in the crop rows and/or the monoculture croplands (Figures 6A–F). For example, in all three soil types, *napA* genes were more abundant in the tree row than in the middle of the crop row of the agroforestry and the monoculture croplands (*p* ≤ 0.031) (Figure 6A). A similar pattern emerged for *nirK*: gene copies of *nirK* were greater in the tree row than in the crop row of the agroforestry and the monoculture croplands in all soil types (*p* ≤ 0.023) (Figure 6C). In the Calcaric Phaeozem and the Gleyic Cambisol, *nirS* gene abundance was 1.9 to 3.0 times greater in the tree row than in the monoculture croplands (*p* ≤ 0.016) (Figure 6D).

### Relationships Between Soil Bacteria, Fungi, N-Cycling Genes and Soil Properties

**FIGURE 7:**
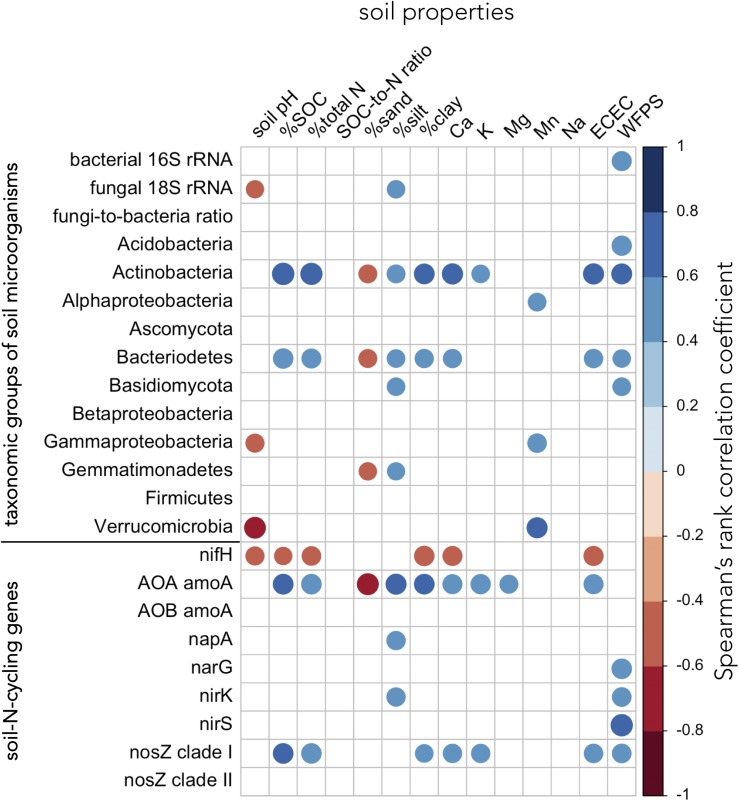
Relationships between groups of soil microorganisms, soil-N-cycling genes, and soil properties. Relationships were assessed using Spearman’s rank correlation test across all replicate plots of paired temperate agroforestry and monoculture croplands in all three soil types (*n* = 60). The diameter of the circles is proportional to the absolute magnitude of the correlation coefficient. Only statistically significant correlations at *p* < 0.05 are shown. AOA = ammonia-oxidizing archaea, AOB = ammonia-oxidizing bacteria, ECEC = effective cation exchange capacity, SOC = soil organic carbon, WFPS = water-filled pore space.

Soil properties associated with soil fertility (SOC, total N, and ECEC) correlated positively with the abundance of Actinobacteria, Bacteriodetes, archaea harboring *amoA*, and bacteria carrying *nosZ* clade I genes (Figure 7 and Supplementary Table 4). Several bacterial (Actinobacteria, Bacteriodetes, and Gemmatimonadetes) and fungal groups (total soil fungi and Basidiomycota) as well as soil-N-cycling genes (AOA *amoA*, *napA*, *nirK*, and *nosZ* clade I) showed positive associations with finer soil texture (silt and/or clay content) (Figure 7 and Supplementary Table 4). Total soil bacteria, the abundance of Acidobacteria, Actinobacteria, Bacteriodetes, and Basidiomycota as well as microorganisms carrying *narG*, *nirK*, *nirS*, and *nosZ* clade I genes were positively correlated to WFPS (Figure 7 and Supplementary Table 4). In contrast, gene abundance of *nifH* was negatively correlated with pH, SOC, total N, clay content, and ECEC (Figure 7 and Supplementary Table 4).

## Discussion

The increased abundance of soil bacteria in the tree rows of the agroforestry systems (Figures 2A, 3) corroborated previous findings of bacterial 16S rRNA abundance in Canadian agroforestry systems (Banerjee et al., 2016). The high WFPS in the tree rows of the agroforestry systems (Supplementary Table 3), a soil property which showed consistent difference from the crop rows and the monoculture croplands across all soil types (Supplementary Table 3), likely contributed to the increased soil bacterial biomass and to the abundance of Acidobacteria, Actinobacteria, and Basidiomycota (Figure 7 and Supplementary Table 4). Furthermore, high WFPS in the tree rows likely increased the abundance of microorganisms harboring *narG*, *nirK*, *nirS*, and *nosZ* clade I genes (Figure 7 and Supplementary Table 4). Changes in soil moisture have been shown to regulate fungal and bacterial population size under field conditions (Schnürer et al., 1986). Likewise, denitrifier abundance has previously been characterized to respond rapidly to manipulations of WFPS (Szukics et al., 2010). Our results were congruent to the findings of Banerjee et al. (2016) who, among other factors (e.g., SOC), attributed greater bacterial 16S rRNA abundance in Canadian agroforestry systems to greater soil moisture in plots with trees. The trees in our agroforestry systems did not increase SOC (Supplementary Table 3), which was probably due to the relatively young age of our agroforestry systems (Lee and Jose, 2003; Pardon et al., 2017). Thus, as opposed to Banerjee et al.’s (2016) findings, the increased soil bacteria at our sites was not attributed to SOC change in the tree rows.

Soil bacterial and fungal biomass have repeatedly been shown to increase with plant biomass and above-ground diversity as well as with the amount and diversity of root exudates (Zak et al., 2003; Steinauer et al., 2016; Eisenhauer et al., 2017; Chen et al., 2019). Therefore, we assumed that the tree litter inputs as well as persistent and abundant tree root biomass and the associated root exudates may have contributed to the promotion of soil bacteria and fungi in the tree rows (Figures 1–3). Furthermore, we observed the existence of an herbaceous layer under the trees of the agroforestry systems (Figure 1D). The biomass of the herbaceous vegetation layer in the tree row was several orders of magnitude smaller than the poplars’ but, in contrast to tree litter, it possesses higher diversity of secondary metabolites (Theis and Lerdau, 2003), which are known to modulate microbial populations (Chomel et al., 2016).

The particularly large increase of Basidiomycota in the tree row in all three soil types (Figure 3B) clearly demonstrated that the trees strongly promoted this fungal group. Since a large proportion of Basidiomycota are wood-decaying and litter-decomposing fungi (Lundell et al., 2014), tree litters (leaves, twigs, roots) accumulating in the tree row (Figure 1D) likely provided Basidiomycota with growth substrate. Similarly, the increased abundance of Ascomycota in the tree row (Figure 4A) may be related to an increase of litter-decomposing members of this phylum (Ma et al., 2013). Additionally, the increased soil moisture under the trees (Supplementary Table 3) was likely to have favored fungal growth (A’Bear et al., 2014). In addition to their function as decomposers, Basidiomycota followed by Ascomycota harbor the majority of ectomycorrhizal fungal lineages (Tedersoo et al., 2010). Therefore, colonization of the poplar root system by ectomycorrhizal fungi likely contributed to the increased abundance of Basidiomycota and Ascomycota in the tree row as compared to the crop row of the agroforestry and monoculture croplands. Furthermore, as tillage is expected to damage hyphal networks (Frey et al., 1999), it was plausible that the absence of tillage in the tree row contributed to the increased soil fungi abundance under the trees (Figures 2B, 3).

The decrease of AOB *amoA* gene copies in the tree row (Figure 5B) was in line with our previous findings of suppression of AOB *amoA* gene abundance by poplar trees (Beule et al., 2019a). High abundance of AOB in cropland samples can likely be accounted for by fertilization, which is common in conventional agriculture as well as in crop rows of the agroforestry systems. Lower abundance of AOB in soil collected below the trees is in line with the fact that trees are not fertilized, which is also a common practice (Tsonkova et al., 2012). Our results further revealed that the increased genetic potential for denitrification in the tree rows as compared to the crop rows of the agroforestry and the monoculture croplands (Figures 6A–F) possibly resulted from the high WFPS in the tree rows (Supplementary Table 3), which enhances denitrification activity (Wen et al., 2017).

Similarly, recent studies indicated that soil moisture and denitrifier abundance are positively linked (Wang et al., 2017; Yang et al., 2018). In line with these findings, the positive correlations of denitrification genes *narG*, *nirK*, *nirS*, and *nosZ* clade I to WFPS (Figure 7 and Supplementary Table 4) suggests that high WFPS in the tree rows (Supplementary Table 3) favored denitrifiers. The greater genetic potential for denitrification in the tree rows corroborates the suggestion by Ferrarini et al. (2017) that short-rotation trees enhance the potential for nitrate removal through denitrification. It should be noted that our study relies on a single sampling time and, thus, does not allow temporal extrapolation. On the other hand, detection of this pattern in one-time sampling warrants quantification of denitrification rates in the field (e.g., using ^15^N_2_O pool dilution techniques), as was done by Wen et al. (2017) in the forests of Lower Saxony, Germany. Combing such *in situ* measurements of denitrification with the quantification of denitrification genes, may be a rewarding research direction to assess the climate-regulation functions of temperate agroforestry systems and conventional systems.

Most soil properties (except for WFPS) within each soil type were not affected by the management system (Supplementary Table 3), which we assume to be due to the relatively young age of our agroforestry systems (Lee and Jose, 2003; Pardon et al., 2017). Therefore, in our correlation analysis, we explored the relationships between microbial groups, N-cycling genes and soil properties across soil types and management systems. The relationship of a high abundance of *nifH* gene with lower soil fertility (i.e., low SOC, total N, and ECEC) (Figure 7 and Supplementary Table 4) contradicted previous studies (Morales et al., 2010; Huhe et al., 2016). These contrasting findings may be explained by the use of different qPCR conditions and primers (Gaby and Buckley, 2012, 2017) or by the contamination of PCR consumables by *nifH*-like DNA, which was reported to occur ubiquitously in certain PCR consumables (Goto et al., 2005). The positive correlations of several microbial groups and N-cycling genes with soil fertility indicators (SOC and total N content and ECEC) and finer soil texture (Figure 7 and Supplementary Table 4) manifested the cyclical associations of these biogeochemical parameters – soil fertility fuels microbial groups and nutrient (e.g., N) cycling genes which, in turn, impact soil available nutrients (Jeffries et al., 2003; van der Heijden et al., 2008; Schlesinger and Bernhardt, 2013).

## Conclusion

Poplar rows in temperate agroforestry systems increased the abundance of several soil bacterial and fungal groups as compared to the crop rows of agroforestry and monoculture croplands. Tree litter input (leaves, twigs, roots) as well as the abundant and persistent tree roots likely contributed to the stimulation of soil microflora under the trees. In addition, the absence of tillage in the tree rows presumably favored fungal communities, particularly Basidiomycota. The poplar rows further promoted the growth of microorganisms harboring denitrification genes, which was likely due to high soil moisture in the tree rows. The higher abundance of denitrification genes suggests that poplar trees may support removal of nitrate from soil via denitrification, and thus minimize nitrate leaching. We suggest that combining our measurements on microbial abundance and N-cycling genes with measurements of soil processes (such as nutrient leaching and soil greenhouse gas fluxes) will enhance insights into the microbial controls of important soil functions of temperate agroforestry systems such as climate regulation and water purification.

## Data Availability Statement

The real-time PCR datasets generated for this study can be found in the BonaRes Centre repository (https://doi.org/10.20387/bonares-9nty-5gfa).

## Author Contributions

LB, MC, MS, EV, and PK contributed to the conception and design of the study. LB and EL conducted the laboratory work and performed the statistical analysis. MS determined the soil properties. LB wrote the first draft of the manuscript. All authors contributed to the manuscript revision, read, and approved the submitted version of the manuscript.

## Conflict of Interest

The authors declare that the research was conducted in the absence of any commercial or financial relationships that could be construed as a potential conflict of interest.
