# Supplementary material for: Poplar Rows in Temperate Agroforestry Croplands Promote Bacteria, Fungi, and Denitrification Genes in Soils
Source: Front Microbiol. 2020 Jan 22;10:3108. doi: 10.3389/fmicb.2019.03108 (PMC6988714; doi:10.3389/fmicb.2019.03108)
Supplement: Supplementary file 1 [file Data_Sheet_1.docx]

Supplementary Material

## Supplementary Figures

**
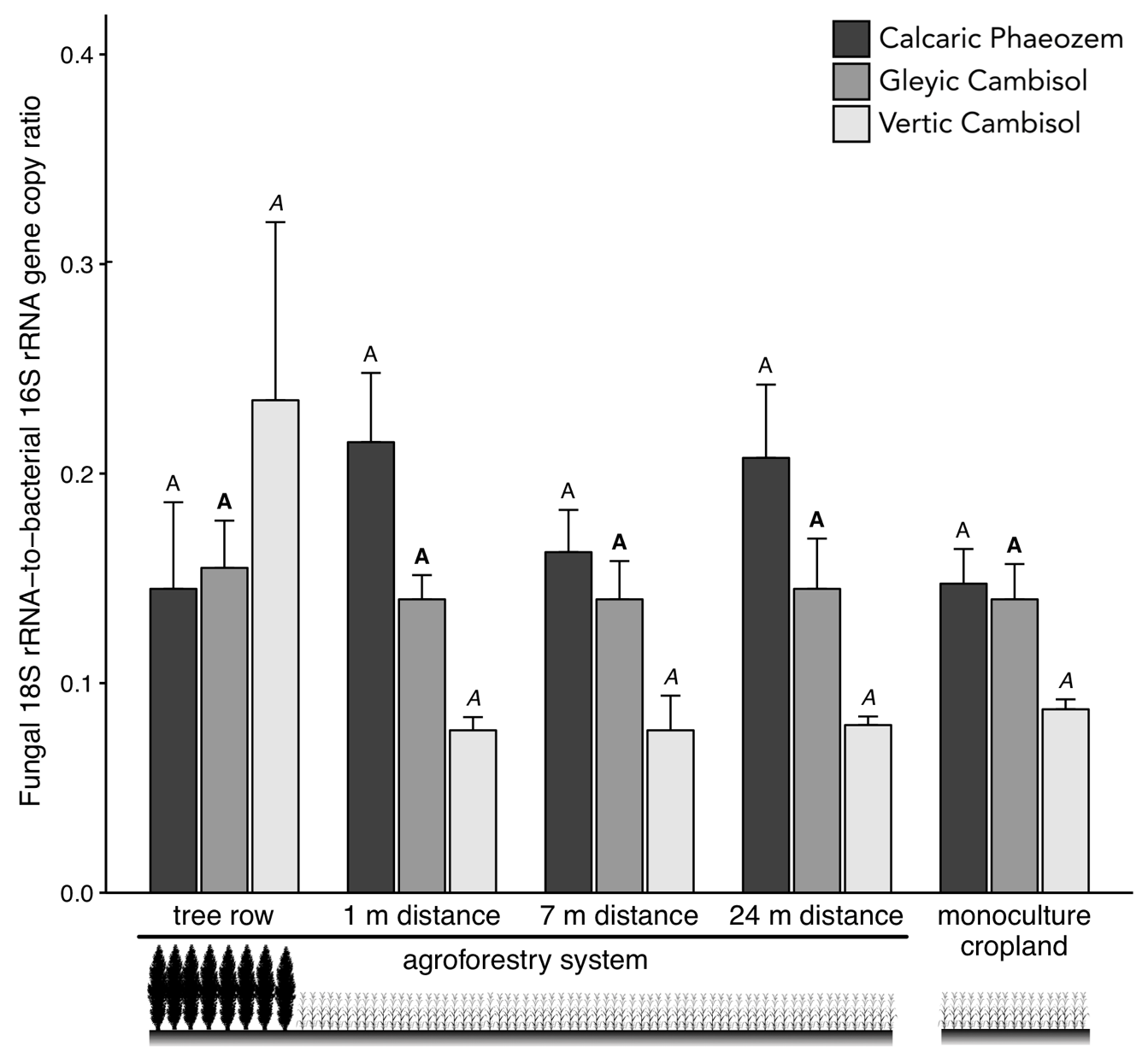
**

**Supplementary Figure 1.** Fungal 18S rRNA-to-bacterial 16S rRNA gene copy ratio in soils of three paired temperate agroforestry and monoculture cropland systems. Means (bars are standard errors; n = 4) with different uppercase letters of the same font indicate statistically significant differences among the sampling locations (the tree row, 1 m, 7 m and 24 m within the crop row of the agroforestry and the monoculture croplands) within one soil type (one-way ANOVA with Tukey’s HSD test of log_10_-transformed data at p < 0.05).


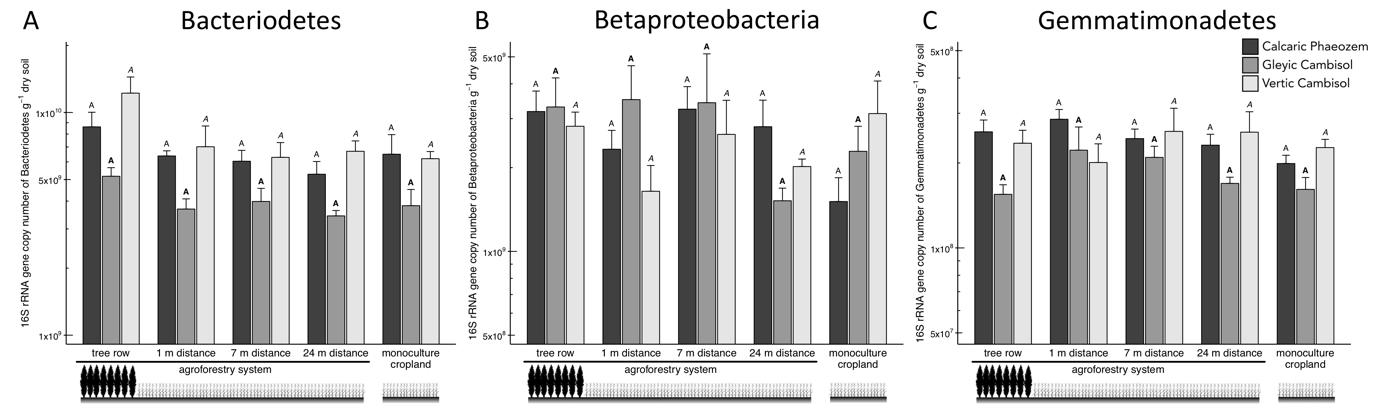


**Supplementary Figure 2.** 16S rRNA gene abundance of three bacterial groups (**(A)** Bacteriodetes, **(B)** Betaproteobacteria, and **(C)** Gemmatimonadetes) in soils of three paired temperate agroforestry and monoculture cropland systems. Means (bars are standard errors; n = 4) with different uppercase letters of the same font indicate statistically significant differences among the sampling locations (the tree row, 1 m, 7 m and 24 m within the crop row of the agroforestry and the monoculture croplands) within one soil type (one-way ANOVA with Tukey’s HSD test of log_10_-transformed data at p < 0.05).

## Supplementary Tables

**Supplementary Table 1.** Primers and standard organisms used for each quantified target microbial group and *nifH*.

| forward primer  (sequence) | reverse Primer  (sequence) | reference | target | organism used for standard |
| --- | --- | --- | --- | --- |
| Eub338  (ACTCCTACGGGAGGCAGCAG) | Eub518  (ATTACCGCGGCTGCTGG) | (Lane, 1991)(Muyzer et al., 1993) | total bacteria | *Pseudomonas stutzeri* ^a^ |
| Acid31  (GATCCTGGCTCAGAATC) | Eub518  (ATTACCGCGGCTGCTGG) | (Barns et al., 1999)(Muyzer et al., 1993) | Acidobacteria | amplified from  environmental DNA |
| Act920F3  (TACGGCCGCAAGGCTA) | Act1200R  (TCRTCCCCACCTTCCTCCG) | (Bacchetti De Gregoris et al., 2011) | Actinobacteria | *Clavibacter michiganensis* ^a^ |
| α682F  (CIAGTGTAGAGGTGAAATT) | 908αR  (CCCCGTCAATTCCTTTGAGTT) | (Bacchetti De Gregoris et al., 2011) | Alphaproteobacteria | *Rhizobium rhizogenes* |
| Cfb319  (GTACTGAGACACGGACCA) | Eub518  (ATTACCGCGGCTGCTGG) | (Muyzer et al., 1993)(Manz et al., 1996) | Bacteriodetes | amplified from  environmental DNA |
| Eub338  (ACTCCTACGGGAGGCAGCAG) | Bet680  (TCACTGCTACACGYG) | (Overmann et al., 1999) | Betaproteobacteria | *Burkholderia cepaciaa* |
| 1080γF  (TCGTCAGCTCGTGTYGTGA) | γ1202R  (CGTAAGGGCCATGATG) | (Bacchetti De Gregoris et al., 2011)(Lane, 1991) | Gammaproteobacteria | *Escheria coli* |
| G1G3-673F  (GAATGCGTAGAGATCC) | 907R  (CCGTCAATTCMTTTRAGTTT) | (DeBruyn et al., 2011)(Lane et al., 1985) | Gemmatimonadetes | amplified from  environmental DNA |
| Lgc353  (GCAGTAGGGAATCTTCCG) | Eub518  (ATTACCGCGGCTGCTGG) | (Meier et al., 1999)(Muyzer et al., 1993) | Firmicutes | *Bacillus licheniformis* |
| Verr349  (GYGGCASCAGKCGMGAAW) | Eub518  (ATTACCGCGGCTGCTGG) | (Philippot et al., 2009)(Muyzer et al., 1993) | Verrucomicrobia | amplified from  environmental DNA |
| FR1  (AICCATTCAATCGGTAIT) | FF390  (CGATAACGAACGAGACCT) | (Vainio and Hantula, 2000) | total fungi | *Verticillium longisporum* ^b^ |
| ITS4Asco  (CGTTACTRRGGCAATCCCTGTTG) | ITS5  (GGAAGTAAAAGTCGTAACAAGG) | (White et al., 1990)(Nikolcheva and Bärlocher, 2004) | Ascomycota | *Fusarium graminearum* |
| ITS4b  (CAGGAGACTTGTACACGGTCCAG) | 5.8sr  (TCGATGAAGAACGCAGCG) | (Gardes and Bruns, 1993; Vilgalys and Hester, 1990) | Basidiomycota | commercial *Agaricus bisporus* |
| IGK3  (GCIWTHTAYGGIAARGGIGGIATHGGIAA) | DVV  (ATIGCRAAICCICCRCAIACIACRTC) | (Ando et al., 2005) | *nifH* | *Azotobacter vinelandii* ^a^ |

^a^ provided by A. Mavridis (University of Goettingen), ^b^ provided by A. von Tiedemann (University of Goettingen).

**Supplementary Table 2.** Real-time PCR conditions for each target microbial group and nifH.

| target | reaction buffer ^a^ | DNA Polymerase | final MgCl_2_ concentration  (mM) | primer concentration  (µM) | initial denaturation | 35 cycles | | |
| --- | --- | --- | --- | --- | --- | --- | --- | --- |
|  |  |  |  |  |  | denaturation | annealing | extension |
| total bacteria | 1X ThermoPol^®^ Reaction Buffer | Taq DNA Polymerase | 2.0 | 0.5 | 95°C, 120s | 94°C, 20s | 57°C, 30s | 68°C, 20s |
| Acidobacteria | 1X ThermoPol^®^ Reaction Buffer | Taq DNA Polymerase | 2.0 | 0.3 | 95°C, 120s | 94°C, 20s | 56°C, 30s | 68°C, 35s |
| Actinobacteria | 1X FastGene^®^ Taq HotStart Buffer | FastGene^®^ Taq DNA Polymerase | 2.0 | 0.3 | 95°C, 120s | 94°C, 20s | 62°C, 30s | 72°C, 20s |
| Alphaproteobacteria | 1X ThermoPol^®^ Reaction Buffer | NEB Taq DNA Polymerase | 2.0 | 0.3 | 95°C, 120s | 94°C, 20s | 60°C, 30s | 68°C, 20s |
| Bacteriodetes | 1X FastGene^®^ Taq HotStart Buffer | FastGene^®^ Taq DNA Polymerase | 2.0 | 0.3 | 95°C, 120s | 94°C, 20s | 62°C, 30s | 72°C, 20s |
| Betaproteobacteria | 1X FastGene^®^ Taq HotStart Buffer | FastGene^®^ Taq DNA Polymerase | 2.0 | 0.3 | 95°C, 120s | 94°C, 20s | 58°C, 30s | 72°C, 25s |
| Gammaproteobacteria | 1X ThermoPol^®^ Reaction Buffer | Taq DNA Polymerase | 2.0 | 0.3 | 95°C, 120s | 94°C, 20s | 60°C, 30s | 68°C, 20s |
| Gemmatimonadetes | 1X ThermoPol^®^ Reaction Buffer | Taq DNA Polymerase | 2.0 | 0.3 | 95°C, 120s | 94°C, 20s | 58°C, 30s | 68°C, 20s |
| Firmicutes | 1X ThermoPol^®^ Reaction Buffer | Taq DNA Polymerase | 2.0 | 0.3 | 95°C, 120s | 94°C, 20s | 57°C, 30s | 68°C, 20s |
| Verrucomicrobia | 1X ThermoPol^®^ Reaction Buffer | Taq DNA Polymerase | 2.0 | 0.3 | 95°C, 120s | 94°C, 20s | 63°C, 30s | 68°C, 20s |
| total fungi | 1X Standard Taq Reaction Buffer | Hot Start Taq DNA Polymerase | 2.5 | 0.3 | 95°C, 120s | 94°C, 20s | 55°C, 30s | 68°C, 30s |
| Ascomycota | 1X Standard Taq Reaction Buffer | Hot Start Taq DNA Polymerase | 1.5 | 0.3 | 95°C, 120s | 94°C, 20s | 55°C, 30s | 68°C, 40s |
| Basidiomycota | 10X Standard Taq Reaction Buffer | Hot Start Taq DNA Polymerase | 1.5 | 0.3 | 95°C, 120s | 94°C, 20s | 59°C, 30s | 68°C, 40s |
| *nifH* | 1X FastGene^®^ Taq HotStart Buffer | FastGene^®^ Taq DNA Polymerase | 2.5 | 0.75 | 95°C, 120s | 94°C, 20s ^b^ | 55°C, 30s ^b^ | 72°C, 30s ^b^ |

^a^ 1X ThermoPol^®^ Reaction Buffer: 20 mM Tris-HCl, 10 mM (NH_4_)_2_SO_4_ ,10 mM KCl, 2 mM MgSO_4_, 0.1% Triton^®^ X-100, pH 8.8 at 25°C (New England Biolabs, Beverly, MA, USA); 1X FastGene^®^ Taq HotStart Buffer (NIPPON Genetics Europe, Düren, Germany); 1X Standard Taq Reaction Buffer: 10 mM Tris-HCl, 50 mM KCl, 1.5 mM MgCl_2_, pH 8.3 at 25°C (New England Biolabs, Beverly, MA, USA). ^b^ including 6 touchdown cycles from 61°C to 56°C.

**Supplementary Table 3.** Soil properties of three paired temperate agroforestry and monoculture cropland systems.

| Soil properties |  | | | | |
| --- | --- | --- | --- | --- | --- |
|  | Calcaric Phaeozem^1^ | | | | |
|  | agroforestry cropland | | | | monoculture cropland |
|  | tree row | 1 m distance^2^ | 7 m distance^2^ | 24 m distance^2^ |  |
| soil pH (in 1:4 H2O) | 6.48 ± 0.07 b | 6.70 ± 0.06 b | 6.73 ± 0.05 b | 6.78 ± 0.02 b | 7.93 ± 0.09 a |
| SOC content (%) | 1.73 ± 0.13 a | 1.46 ± 0.03 a | 1.35 ± 0.07 a | 1.51 ± 0.03 a | 1.30 ± 0.21 a |
| total N content (%) | 0.18 ± 0.01 a | 0.17 ± 0.00 a | 0.16 ± 0.01 a | 0.17 ± 0.01 a | 0.16 ± 0.02 a |
| SOC-to-N ratio | 9.58 ± 0.34 a | 8.70 ± 0.17 ab | 8.58 ± 0.14 ab | 8.93 ± 0.34 ab | 8.13 ± 0.41 b |
| sand content (%) | 3.75 ± 0.25 b | 4.50 ± 0.29 ab | 4.00 ± 0.00 ab | 4.00 ± 0.00 ab | 10.75 ± 1.31 a |
| silt content (%) | 75.75 ± 0.85 a | 73.00 ± 2.42 a | 72.50 ± 1.85 a | 68.75 ± 1.70 a | 51.25 ± 3.15 b |
| clay content (%) | 20.50 ± 1.04 b | 22.50 ± 2.60 b | 23.50 ± 1.85 b | 27.25 ± 1.70 b | 38.00 ± 1.91 a |
| exchangeable Ca (mmolc kg^-1^) | 128.51 ± 3.39 b | 133.98 ± 1.62 b | 138.18 ± 3.04 b | 138.87 ± 3.26 b | 550.96 ± 102.69 a |
| exchangeable K (mmolc kg^-1^) | 8.75 ± 0.93 a | 8.17 ± 0.87 a | 8.09 ± 0.45 a | 8.10 ± 0.49 a | 9.76 ± 1.10 a |
| exchangeable Mg (mmolc kg^-1^) | 13.15 ± 0.65 a | 12.60 ± 0.39 a | 13.08 ± 0.55 a | 13.09 ± 0.94 a | 28.38 ± 2.31 a |
| exchangeable Mn (mmolc kg^-1^) | 1.21 ± 0.13 a | 0.70 ± 0.11 ab | 0.55 ± 0.11 ab | 0.60 ± 0.09 ab | 0.08 ± 0.02 b |
| exchangeable Na (mmolc kg^-1^) | 0.55 ± 0.14 a | 0.63 ± 0.07 a | 0.75 ± 0.07 a | 0.51 ± 0.10 a | 0.79 ± 0.17 a |
| ECEC (mmolc kg^-1^) | 152.23 ± 4.71 b | 157.45 ± 2.89 b | 160.70 ± 3.03 b | 161.18 ± 4.32 b | 590.00 ± 100.77 a |
| WFPS (%) | 47.86 ± 1.20 a | 41.54 ± 1.96 b | 29.69 ± 1.37 c | 33.04 ± 1.21 c | 30.23 ± 0.99 c |
|  |  | | | | |
|  | Gleyic Cambisol^1^ | | | | |
|  | agroforestry cropland | | | | monoculture cropland |
|  | tree row | 1 m distance^2^ | 7 m distance^2^ | 24 m distance^2^ |  |
| soil pH (in 1:4 H2O) | 7.05 ± 0.13 a | 7.05 ± 0.09 a | 7.00 ± 0.07 a | 7.00 ± 0.08 a | 7.08 ± 0.02 a |
| SOC content (%) | 1.08 ± 0.07 a | 1.08 ± 0.06 a | 1.12 ± 0.08 a | 1.06 ± 0.05 a | 0.91 ± 0.05 a |
| total N content (%) | 0.12 ± 0.00 a | 0.12 ± 0.01 a | 0.12 ± 0.00 a | 0.11 ± 0.00 a | 0.11 ± 0.00 a |
| SOC-to-N ratio | 8.83 ± 0.72 a | 9.23 ± 0.21 a | 9.30 ± 0.51 a | 9.45 ± 0.30 a | 8.45 ± 0.16 a |
| sand content (%) | 60.75 ± 4.17 a | 66.5 ± 2.63 a | 65.25 ± 2.66 a | 68.5 ± 1.55 a | 66.75 ± 2.9 a |
| silt content (%) | 32.25 ± 3.57 a | 25.5 ± 2.10 a | 24.75 ± 4.64 a | 21.75 ± 1.11 a | 23.75 ± 2.25 a |
| clay content (%) | 7.00 ± 0.71 a | 8.00 ± 1.78 a | 10.00 ± 2.04 a | 9.75 ± 2.21 a | 9.50 ± 1.76 a |
| exchangeable Ca (mmolc kg^-1^) | 46.11 ± 5.14 a | 45.70 ± 6.41 a | 50.61 ± 3.58 a | 42.36 ± 4.05 a | 42.19 ± 6.09 a |
| exchangeable K (mmolc kg^-1^) | 1.44 ± 0.43 a | 1.20 ± 0.48 a | 1.55 ± 0.54 a | 1.21 ± 0.28 a | 0.67 ± 0.13 a |
| exchangeable Mg (mmolc kg^-1^) | 9.75 ± 1.87 a | 8.38 ± 1.80 a | 9.26 ± 0.78 a | 8.61 ± 1.29 a | 10.76 ± 0.99 a |
| exchangeable Mn (mmolc kg^-1^) | 0.30 ± 0.11 a | 0.29 ± 0.04 a | 0.29 ± 0.05 a | 0.26 ± 0.05 a | 0.18 ± 0.01 a |
| exchangeable Na (mmolc kg^-1^) | 0.29 ± 0.08 a | 0.28 ± 0.18 a | 0.59 ± 0.18 a | 0.30 ± 0.18 a | 0.32 ± 0.09 a |
| ECEC (mmolc kg^-1^) | 57.93 ± 7.06 a | 55.80 ± 8.26 a | 62.38 ± 3.32 a | 52.80 ± 5.34 a | 54.30 ± 6.52 a |
| WFPS (%) | 45.30 ± 4.24 a | 40.61 ± 2.23 ab | 34.69 ± 1.86 ab | 33.95 ± 2.70 ab | 31.50 ± 1.64 b |
|  |  | | | | |
|  | Vertic Cambisol^1^ | | | | |
|  | agroforestry cropland | | | | monoculture cropland |
|  | tree row | 1 m distance^2^ | 7 m distance^2^ | 24 m distance^2^ |  |
| soil pH (in 1:4 H2O) | 7.08 ± 0.26 a | 7.43 ± 0.13 a | 7.30 ± 0.22 a | 7.33 ± 0.09 a | 7.43 ± 0.02 a |
| SOC content (%) | 2.56 ± 0.13 a | 2.51 ± 0.05 a | 2.48 ± 0.05 a | 2.40 ± 0.03 a | 2.31 ± 0.04 a |
| total N content (%) | 0.27 ± 0.01 a | 0.26 ± 0.01 a | 0.26 ± 0.01 a | 0.26 ± 0.00 a | 0.27 ± 0.02 a |
| SOC-to-N ratio | 9.65 ± 0.12 a | 9.58 ± 0.13 a | 9.53 ± 0.07 a | 9.25 ± 0.10 a | 8.65 ± 0.46 a |
| sand content (%) | 18.25 ± 2.98 a | 16.25 ± 3.17 a | 20.5 ± 3.66 a | 16.75 ± 2.93 a | 27.00 ± 2.04 a |
| silt content (%) | 46.75 ± 3.64 a | 46.75 ± 3.30 a | 41.75 ± 2.14 ab | 43.50 ± 4.57 ab | 28.50 ± 4.17 b |
| clay content (%) | 35.00 ± 2.42 a | 37.00 ± 1.08 a | 37.75 ± 3.52 a | 39.75 ± 3.84 a | 44.50 ± 2.72 a |
| exchangeable Ca (mmolc kg^-1^) | 339.64 ± 78.61 a | 359.10 ± 77.75 a | 374.97 ± 97.34 a | 357.60 ± 88.62 a | 288.89 ± 8.62 a |
| exchangeable K (mmolc kg^-1^) | 7.12 ± 0.11 a | 7.36 ± 0.42 a | 7.48 ± 0.52 a | 7.42 ± 0.34 a | 7.94 ± 0.62 a |
| exchangeable Mg (mmolc kg^-1^) | 14.93 ± 2.15 a | 13.86 ± 1.50 a | 13.90 ± 1.58 a | 13.89c 1.57 a | 17.92 ± 0.94 a |
| exchangeable Mn (mmolc kg^-1^) | 0.31 ± 0.04 a | 0.19 ± 0.06 a | 0.18 ± 0.03 a | 0.16 ± 0.04 a | 0.17 ± 0.01 a |
| exchangeable Na (mmolc kg^-1^) | 0.51 ± 0.14 a | 0.47 ± 0.05 a | 0.56 ± 0.05 a | 0.71 ± 0.10 a | 0.58 ± 0.17 a |
| ECEC (mmolc kg^-1^) | 362.55 ± 76.70 a | 381.05 ± 76.07 a | 397.15 ± 95.79 a | 379.80 ± 87.17 a | 315.55 ± 7.71 a |
| WFPS (%) | 62.64 ± 3.25 a | 45.19 ± 1.07 b | 44.71 ± 1.43 b | 41.94 ± 0.73 b | 42.26 ± 0.60 b |

Soil pH, SOC and total N content, SOC-to-N ratio, sand, silt, and clay content, exchangeable Ca, K, Mg, Mn, and Na were measured in the top 0.0-0.3 m soil depth in 2016, water-filled pore space (WFPS) was measured in the top 0.0-0.005 m soil depth in spring 2019 on the same day that soil samples for DNA extraction were collected. Soil data are provided by Schmidt et al. (unpublished data). ^1^ Means ± standard error (n = 4) followed by a different lowercase letter indicate significant differences among sampling locations within the agroforestry and the monoculture croplands (one-way ANOVA with Tukey’s HSD test or Kruskal-Wallis test with multiple comparison extension at p < 0.05). ^2^ Distance within the crop row from the tree row of the agroforestry system.

**Supplementary Table 4.** Spearman’s rank correlation coefficient of the relationships of soil microbial groups, soil-N-cycling genes and soil properties.

|  | soil pH  (in 1:4 H2O) | SOC content (%) | Total N (%) | SOC-to-N ratio | Sand content (%) | Silt content (%) | Clay content (%) | exchangeable Ca  (mmolc kg^-1^) | exchangeable K  (mmolc kg^-1^) | exchangeable Mg  (mmolc kg^-1^) | exchangeable Mn  (mmolc kg^-1^) | exchangeable Na  (mmolc kg^-1^) | ECEC  (mmolc kg^-1^) | WFPS (%) |
| --- | --- | --- | --- | --- | --- | --- | --- | --- | --- | --- | --- | --- | --- | --- |
| bacterial 16S rRNA genes | -0.28 | 0.33 | 0.31 | 0.17 | -0.40 | 0.45 | 0.17 | 0.17 | 0.3 | 0.2 | 0.29 | 0.01 | 0.18 | **0.57***** |
| fungal 18S rRNA gees | **-0.47*** | -0.08 | -0.11 | -0.05 | -0.38 | **0.48*** | -0.20 | -0.09 | 0.17 | -0.06 | 0.46 | -0.05 | -0.09 | 0.23 |
| fungi-to-bacteria ratio | -0.38 | -0.43 | -0.45 | -0.29 | -0.12 | 0.22 | -0.43 | -0.32 | -0.01 | -0.23 | 0.39 | -0.07 | -0.32 | -0.23 |
| Acidobacteria | -0.23 | 0.30 | 0.29 | 0.21 | -0.35 | 0.43 | 0.16 | 0.20 | 0.29 | 0.22 | 0.27 | 0.05 | 0.20 | 0.58*** |
| Actinobacteria | 0.02 | **0.71***** | **0.70***** | 0.25 | -0.50 | **0.50*** | **0.61***** | **0.64***** | **0.48*** | 0.41 | 0.02 | 0.17 | **0.64***** | **0.62***** |
| Alphaproteobacteria | -0.31 | 0.09 | 0.09 | 0.04 | **-0.41*** | 0.46 | 0.03 | 0.08 | 0.31 | 0.21 | **0.49*** | 0.1 | 0.09 | 0.33 |
| Ascomycota | -0.40 | -0.18 | -0.19 | -0.09 | -0.30 | 0.39 | -0.20 | -0.10 | 0.1 | 0.04 | 0.43 | 0.01 | -0.10 | 0.14 |
| Bacteriodetes | -0.03 | **0.59***** | **0.56***** | 0.24 | **-0.50*** | **0.49*** | **0.52**** | **0.52**** | 0.44 | 0.34 | 0.01 | 0.21 | **0.53**** | **0.48*** |
| Basidiomycota | -0.41 | 0.09 | 0.03 | 0.27 | -0.34 | **0.48*** | -0.17 | -0.04 | 0.05 | -0.07 | 0.30 | 0.09 | -0.04 | **0.49*** |
| Betaproteobacteria | -0.33 | 0.03 | 0.02 | 0.05 | -0.11 | 0.15 | -0.08 | -0.11 | -0.07 | 0.01 | 0.30 | -0.03 | -0.10 | 0.14 |
| Gammaproteobacteria | **-0.51**** | -0.12 | -0.12 | -0.08 | -0.30 | 0.39 | -0.27 | -0.23 | 0.08 | -0.08 | **0.54**** | -0.09 | -0.23 | 0.16 |
| Gemmatimonadetes | -0.34 | 0.34 | 0.34 | 0.01 | **-0.47*** | **0.46*** | 0.29 | 0.26 | 0.4 | 0.31 | 0.35 | 0.14 | 0.26 | 0.18 |
| Firmicutes | -0.37 | 0.01 | -0.01 | 0.03 | -0.37 | 0.44 | -0.08 | -0.04 | 0.14 | 0.03 | 0.39 | -0.08 | -0.03 | 0.29 |
| Verrucomicrobia | **-0.67***** | -0.17 | -0.21 | 0.08 | -0.20 | 0.31 | -0.41 | -0.38 | -0.08 | -0.19 | **0.63***** | -0.17 | -0.38 | 0.35 |
| *nifH* | **-0.52**** | **-0.47*** | **-0.54**** | -0.01 | 0.14 | -0.06 | **-0.59***** | **-0.57***** | -0.41 | -0.42 | 0.37 | -0.11 | **-0.57***** | -0.17 |
| AOA *amoA* | -0.14 | **0.61***** | **0.60***** | 0.09 | **-0.68***** | **0.63***** | **0.60***** | **0.57***** | **0.58***** | **0.51*** | 0.17 | 0.21 | **0.57***** | 0.35 |
| AOB *amoA* | 0.25 | 0.06 | 0.03 | -0.06 | -0.03 | -0.08 | 0.22 | 0.20 | 0.07 | 0.07 | -0.27 | 0.03 | 0.21 | -0.29 |
| *napA* | -0.36 | 0.09 | 0.09 | 0.03 | -0.44 | **0.52**** | -0.01 | 0.07 | 0.27 | 0.12 | 0.36 | 0.07 | 0.07 | 0.37 |
| *narG* | -0.03 | 0.34 | 0.34 | 0.23 | -0.17 | 0.24 | 0.17 | 0.24 | 0.14 | 0.18 | 0.13 | -0.19 | 0.25 | **0.60***** |
| *nirK* | -0.29 | 0.31 | 0.31 | 0.17 | -0.45 | **0.52**** | 0.15 | 0.22 | 0.32 | 0.23 | 0.33 | -0.01 | 0.22 | **0.54**** |
| *nirS* | -0.15 | 0.36 | 0.33 | 0.39 | -0.17 | 0.25 | 0.18 | 0.20 | 0.14 | 0.25 | 0.19 | -0.03 | 0.20 | **0.71***** |
| *nosZ* clade I | 0.10 | **0.60***** | **0.60***** | 0.26 | -0.35 | 0.38 | **0.49*** | **0.53**** | **0.53**** | 0.46 | 0.04 | 0.12 | **0.54**** | **0.54**** |
| *nosZ* clade II | -0.42 | 0.18 | 0.16 | 0.22 | -0.11 | 0.15 | -0.06 | -0.17 | -0.07 | -0.03 | 0.38 | -0.15 | -0.17 | 0.33 |

Statistically significant correlations are shown bold (Spearman’s rank correlation test). * p < 0.05, ** p < 0.01, ** p < 0.001. SOC = soil organic carbon, ECEC = effective cation exchange capacity, WFPS = water-filled pore space.
